# AI Meets Exascale Computing: Advancing Cancer Research With Large-Scale High Performance Computing

**DOI:** 10.3389/fonc.2019.00984

**Published:** 2019-10-02

**Authors:** Tanmoy Bhattacharya, Thomas Brettin, James H. Doroshow, Yvonne A. Evrard, Emily J. Greenspan, Amy L. Gryshuk, Thuc T. Hoang, Carolyn B. Vea Lauzon, Dwight Nissley, Lynne Penberthy, Eric Stahlberg, Rick Stevens, Fred Streitz, Georgia Tourassi, Fangfang Xia, George Zaki

**Affiliations:** ^1^Theoretical Division, Los Alamos National Laboratory, Los Alamos, NM, United States; ^2^Computing, Environment and Life Sciences Directorate, Argonne National Laboratory, Lemont, IL, United States; ^3^Division of Cancer Treatment and Diagnosis, National Cancer Institute, Bethesda, MD, United States; ^4^Applied Development and Research Directorate, Frederick National Laboratory for Cancer Research, Frederick, MD, United States; ^5^Center for Biomedical Informatics and Information Technology, National Cancer Institute, Bethesda, MD, United States; ^6^Physical and Life Sciences Directorate, Lawrence Livermore National Laboratory, Livermore, CA, United States; ^7^National Nuclear Security Administration, U.S. Department of Energy, Advanced Simulation and Computing, Washington, DC, United States; ^8^Office of Science, U.S. Department of Energy, Advanced Scientific Computing Research, Washington, DC, United States; ^9^NCI RAS Initiative, Cancer Research Technology Program, Frederick National Laboratory for Cancer Research, Frederick, MD, United States; ^10^Division of Cancer Control and Population Sciences, National Cancer Institute, Bethesda, MD, United States; ^11^Biomedical Informatics and Data Science Directorate, Frederick National Laboratory for Cancer Research, Frederick, MD, United States; ^12^Computer Science Department, University of Chicago, Chicago, IL, United States; ^13^High Performance Computing Innovation Center, Lawrence Livermore National Laboratory, Livermore, CA, United States; ^14^Health Data Sciences Institute, Oak Ridge National Laboratory, Oak Ridge, TN, United States; ^15^Data Science and Learning Division, Argonne National Laboratory, Lemont, IL, United States

**Keywords:** cancer research, high performance computing, artificial intelligence, deep learning, natural language processing, multi-scale modeling, precision medicine, uncertainty quantification

## Abstract

The application of data science in cancer research has been boosted by major advances in three primary areas: (1) Data: diversity, amount, and availability of biomedical data; (2) Advances in Artificial Intelligence (AI) and Machine Learning (ML) algorithms that enable learning from complex, large-scale data; and (3) Advances in computer architectures allowing unprecedented acceleration of simulation and machine learning algorithms. These advances help build *in silico* ML models that can provide transformative insights from data including: molecular dynamics simulations, next-generation sequencing, omics, imaging, and unstructured clinical text documents. Unique challenges persist, however, in building ML models related to cancer, including: (1) access, sharing, labeling, and integration of multimodal and multi-institutional data across different cancer types; (2) developing AI models for cancer research capable of scaling on next generation high performance computers; and (3) assessing robustness and reliability in the AI models. In this paper, we review the National Cancer Institute (NCI) -Department of Energy (DOE) collaboration, *Joint Design of Advanced Computing Solutions for Cancer (JDACS4C)*, a multi-institution collaborative effort focused on advancing computing and data technologies to accelerate cancer research on three levels: molecular, cellular, and population. This collaboration integrates various types of generated data, pre-exascale compute resources, and advances in ML models to increase understanding of basic cancer biology, identify promising new treatment options, predict outcomes, and eventually prescribe specialized treatments for patients with cancer.

## Introduction

Predictive computational models for patients with cancer can in the future support prevention and treatment decisions by informing choices to achieve the best possible clinical outcome. Toward this vision, in 2015, the national Precision Medicine Initiative (PMI) (1) was announced, motivating efforts to target and advance precision oncology, including looking ahead to the scientific, data and computational capabilities needed to advance this vision. At the same time, the horizon of computing was changing in the life sciences, as the capabilities and transformations enabled by exascale computing were coming into focus, driven by the accelerated growth in data volumes and anticipated new sources of information catalyzed by new technologies and initiatives such as PMI.

The National Strategic Computing Initiative (NSCI) in 2015 named the Department of Energy (DOE) as a lead agency for “advanced simulation through a capable exascale computing program” and the National Institutes of Health (NIH) as one of the deployment agencies to participate “in the co-design process to integrate the special requirements of their respective missions.” This interagency coordination structure opened the avenue for a tight collaboration between the NCI and the DOE. With shared aims to advance cancer research while shaping the future for exascale computing, the NCI and DOE established the JDACS4C in June of 2016 through a 5-year memorandum of understanding with three co-designed pilot efforts to address both national priorities. The high-level goals of these three pilots were to push the frontiers of computing technologies in specific areas of cancer research: (1) Cellular-level: advance the capabilities of patient-derived pre-clinical models to identify new treatments; (2) Molecular-level: further understand the basic biology of undruggable targets; and (3) Population-level: gain critical insights on the drivers of population cancer outcomes. The pilots would also develop new Uncertainty Quantification (UQ) methods to evaluate confidence in the AI model predictions.

Using co-design principles, each of the pilots in the JDACS4C collaboration is based on—and driven by—team science, which is the hallmark of the collaboration's success. Enabled by deep learning, Pilot One (cellular-level) combines data in innovative ways to develop computationally predictive models for tumor response to novel therapeutic agents. Pilot Two (molecular-level) combines experimental data, simulation, and AI to provide new windows to understand and explore the biology of RAS-related cancers. Pilot Three (population-level) uses AI and clinical information at unprecedented scales to enable precision cancer surveillance to transform cancer care.

## AI and Large-Scale Computing to Predict Tumor Treatment Response

**Figure 1:**
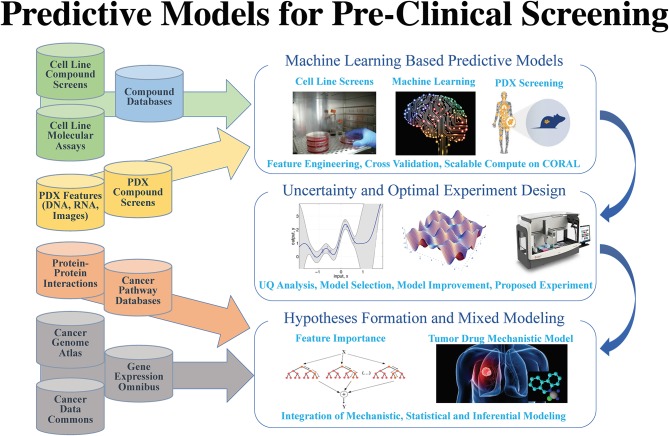
Pilot 1 research aims, general workflow, and supporting data.

After years of efforts within the research and pharmaceutical sectors, many patients with cancer still do not respond to standard-of-care treatments, and emergence of therapy resistance is common. Efforts in precision medicine may someday change this by using a targeted therapeutics approach, individually tailored to each patient based on predictive models that use molecular and drug signatures. The *Predictive Modeling for Pre-Clinical Screening Pilot* (Pilot One) aims to develop predictive capabilities of drug response in pre-clinical models of cancer to improve and expedite the selection and development of new targeted therapies for patients with cancer. Highlights of the work done in Pilot One is shown in Figure 1.

As omics data continues to accumulate, computational models integrating multimodal data sources become possible. Multimodal deep learning (2) aims to enhance learned features for one task by learning features over multiple modalities. Early Pilot One work (3) measured performance of multi-modal deep neural network drug pair response models with 5-fold cross validation. Using the NCI-ALMANAC (4) data, best model performance was demonstrated when gene expression, microRNA, proteome, and Dragon7 drug descriptors (5) were combined obtaining an R-squared value of 0.944, which indicates that over 94% of the variation in tumor response is explained by the variation among the contributing gene expression, micro RNA expression, proteomics and drug property data.

Mechanistically informed feature selection is an alternative approach that has the potential to increase predictive model performance. The LINCS landmark genes (6) for example has been used to train deep learning models to predict gene expression of non-landmark genes (7) and to classify drug-target interactions (8). Ongoing work in Pilot One is exploring the impact on prediction using gene sets like that of the LINCS landmark genes and other mechanistically defined gene sets. The potential of employing mechanistically informed feature selection extends beyond improving prediction accuracy, to building models on the basis of existing biological knowledge.

Transfer learning is another area of important research activity. The goal of transfer learning is to improve learning in the target learning task by leveraging knowledge from an existing source task (9). Given challenges in obtaining sufficient data for target Patient Derived Xenografts (PDXs), where tumors are grown in mouse host animals, ongoing transfer learning work holds promise for learning on cell lines as a source for the target PDX model predictions. Pilot One is first working on generating models that generalize across cell line studies, a precursor to transfer learning from cell lines to PDXs.

Using data from the NCI-ALMANAC (4), NCI-60 (10), GDSC (11), CTRP (12), gCSI (13), and CCLE (14), models can be constructed that generalize across cell-line studies. Using multi-task networks which combines additional learning of three different classification tasks—tumor/normal, cancer type, and cancer site—with learning of the drug response task, it could be possible to capture more of the total variance and improve precision and recall when training on CTRP and predicting on CCLE for example. Demonstrating cross-study model capability will provide additional confidence that general models can be developed for prediction tasks on cell lines and PDXs and organoids.

Answering questions of how much data and what methods are suitable is a critical part of Pilot One. Although it is generally held that deep learning methods outperform traditional machine learning methods when large data sets are used, this has not yet been explored in the context of drug response prediction problem. Early efforts underway in Pilot One are exploring the relationship among sample size, deep learning methods, and traditional machine learning methods to better characterize the dependencies on predictive performance. This information of sample size together with model accuracy metrics will be of critical importance to future experimental designs for those who wish to pursue deep learning approaches to the drug response prediction problem.

Such extensive deep learning and machine learning investigations require significant computational resources, such as those available at DOE Leadership Computing Facilities (LCF) employed by Pilot 1. A recent experiment searched 23,200 deep neural network models using COXEN (15) selected features and Bayesian optimization ideas (16) to find the best model hyperparameters (hyperparameters generally define the choice of functions and relationship among functions in a given deep learning model). This produced the best cross-study validation results to-date, underscoring the critical need for feature selection and hyperparameter optimization when building predictive models. Further, uncertainty quantification (explained in more depth later) adds a new level of computing demand. Uncertainty quantification experiments involving over 30 billion predictions from 450 of the best models generated on the DOE Summit LCF system are ongoing to understand the relationship to between best model uncertainty and the model that performs best in cross-study validation experiments.

Reflecting on insights from Pilot 1 activities and current gaps in available literature, future work will focus on exploring new predictive models to better utilize, ground, and enrich biological knowledge. Efforts to improve drug representations for response prediction are expected to benefit from research involving training semi-supervised networks on millions of compounds. In efforts to improve understanding of trained models, mechanistic information is being incorporated into more interpretable deep learning models. Active learning in response prediction—which balances uncertainty, accuracy, and lead discovery—will be used to guide the acquisition of experimental data for animal models in a cost-effective and timely manner. And finally, a necessary step toward precision models is gaining a fine-grained understanding of prediction error, an insight enabled by the demonstrated capability in large-scale model sweeps.

## AI at the Forefront of RAS Related Cancers

Oncogenic mutations in RAS genes are associated with more than 30% of cancers and are particularly prevalent in those of the lung, colon and pancreas. Though RAS mutations have been studied for decades, there are currently no RAS inhibitors and a detailed molecular mechanism for how RAS engages and activates proximal signaling proteins (RAF) remains elusive (17). RAS signaling takes place at and is dependent on cellular membranes, a complex cellular environment that is difficult to recapitulate using current experimental technologies.

**Figure 2:**
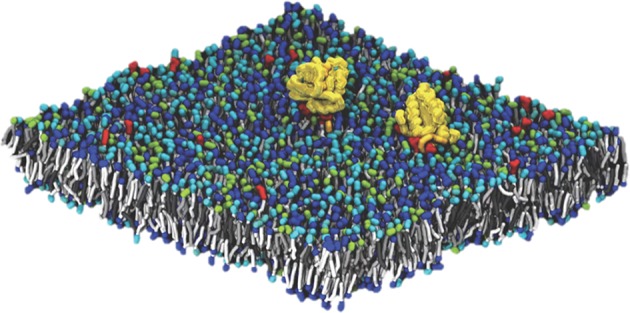
CGMD simulation captures the molecular details of RAS in complex lipid membranes.

Pilot Two*, Improving Outcomes for RAS-related Cancer*, is focused on delivering a validated multiscale model of RAS biology on a cell membrane by combining the experimental capabilities at the Frederick National Laboratory for Cancer Research with the computational resources of the National Nuclear Security Administration (NNSA), a semi-autonomous agency of the DOE. The principal challenge in modeling this system is the diverse length and time scales involved. Lipid membranes evolve over a macroscopic scale (micrometers and milliseconds). Capturing this evolution is critical, as changes in lipid concentration define the local environment in which RAS operates. The RAS protein itself, however, binds over time and length scales which are microscopic (nanometers and microseconds). In order to elucidate the behavior of RAS proteins in the context of a realistic membrane, our modeling effort must span the multiple orders of magnitude between microscopic and macroscopic behavior. The Pilot Two team has built such a framework, developing a macroscopic model that captures the evolution of the lipid environment and which is consistent with an optimized microscopic model that captures protein-protein and protein-lipid interactions at the molecular scale. Macroscopic model components (lipid environment, lipid-lipid interactions, protein behavior and protein-lipid interactions) were characterized through close collaboration between the experimentalists at Frederick National Laboratory and the computational scientists from the DOE/NNSA. The microscopic model is based on standard Martini force fields for Coarse-Grained Molecular Dynamics (CGMD), modified to correctly capture certain details of lipid phase behavior (18–21). A snapshot from a typical micro-scale simulation run, showing two RAS proteins on a 30 nm × 30 nm patch of lipid membrane (containing ~150,000 particles) is shown in Figure 2.

In order to bring the two scales together, the team devised a novel workflow whereby microscopic subsections of a running macroscopic model are scored for uniqueness using a machine learning algorithm operating in a reduced order space that has been trained on previous simulations. The most unique subsections in the macroscopic simulation are identified and re-created as CGMD simulations, which explore the microscopic behavior. Information from the (many thousands of) microscopic simulations is then fed back into the macroscopic model, so that it is continually improving even as the simulations are running (22).

This modeling infrastructure was designed to exploit the Sierra supercomputer at Lawrence Livermore National Laboratory. The scale and heterogeneous architecture of Sierra make it ideal for such a workflow that combines AI technology with predictive simulation. Running on the entire machine, the team was able to simulate at the macroscopic level a 1 by 1μm, 14-lipid membrane with 300 RAS proteins, generating over 100,000 microscopic simulations capturing over 200 ms of protein behavior. This unprecedented achievement represents an almost two orders of magnitude improvement on the previously state of the art. That being said, the space of all possible lipid mixtures is huge, requiring tens of thousands of samples for any meaningful coverage. This type of MUltiple Metrics Modeling Infrastructure (MuMMI) simulations will always be limited by the available High Performance Computing (HPC) resources. With an exascale machine we can substantially increase the dimensionality of the input space and its coverage, significantly improving the applicability of future campaigns.

In the coming years, the team will exploit this capability to explore RAS behavior on lipid membranes and extend the model in three important directions. First, both the macro and micro models will be modified to incorporate the RAF kinase, which binds to RAS as the first step in the MAPK pathway that leads to growth signaling. Second, we will extend the infrastructure to include fully atomistic resolution, creating a three-level (macro/micro/atomistic) multiscale model. Third, we will incorporate membrane curvature into the dynamics of the membrane, which is currently constrained to remain flat. The improved infrastructure will allow the largest and most accurate computational exploration of RAS biology to date.

## Advancing Cancer Surveillance Using AI and High-Performance Computing

The Surveillance, Epidemiology, and End Results (SEER) program funded by the NCI was established in 1973 for the advancement of public health and for reducing the cancer burden in the United States. SEER currently collects and publishes cancer incidence and survival data from population-based cancer registries covering ~34.6% of the U.S. population. The curated, population-level SEER data provide a rich information source for data-driven discovery to understand drivers of cancer outcomes in the real world.

An outstanding challenge of the SEER program is how to achieve near real-time cancer surveillance. Information abstraction is a critical step to facilitate data-driven explorations. However, the process is fully manual to ensure high quality data. As the SEER program increases the breadth of information captured, the manual process is no longer scalable. By partnering computational and data scientists from DOE with NCI SEER domain experts, Pilot Three, *Population Information Integration, Analysis, and Modeling for Precision Surveillance*, aims to leverage high-performance computing and artificial intelligence to meet the emerging needs of cancer surveillance. Moreover, Pilot Three envisions a fully integrated data-driven modeling and simulation framework to enable meaningful translation of big SEER data. By collecting and linking additional patient data, we can generate profiles for patients with cancer that include information about healthcare delivery system parameters and continuity of care. Such rich data will facilitate data-driven modeling and simulation of patient-specific health trajectories to support precision oncology research at the population level.

To date, Pilot Three has mainly focused on the development, scaling, and deployment of cutting-edge AI tools to semi-automate information abstraction from unstructured pathology text reports, the main source of information of cancer registries. In partnership with the Louisiana Tumor Registry and the Kentucky Cancer Registry, several AI-based Natural Language Processing (NLP) tools have been developed and benchmarked for abstraction of fundamental cancer data elements such as cancer site, laterality, behavior, histology, and grade (23–29). The NLP tools rely on the latest AI advances including multi-task learning and attention mechanisms. Scalable training and hyperparameter optimization of the tools is managed by relying on pre-exascale computing infrastructure available within the DOE laboratory complex (30). Following an iterative optimization protocol, the most computationally efficient and clinically effective tools are deployed for evaluation across participating SEER registries. Based on preliminary testing the NLP tools have been able to accurately classify all five data elements for 42.5% of cancer cases. Further refinement of this accuracy level is underway in subsequent versions as well as incorporation of an uncertainty quantification component to ease and increase user confidence.

Although the patient information currently collected across SEER registries is mainly clinical (clin-omics), increasingly other -omics type of information is expected to become part of cancer surveillance. Specifically, radiomics (i.e., biomarkers automatically extracted from histopathological and radiological images via targeted image processing algorithms) as well as genomics will provide important insight to understand the effectiveness of cancer treatment choices.

Moving forward, Pilot Three will implement the latest NLP tools into production application across participating SEER registries using Application Program Interfaces (APIs) to determine the most effective human-AI workflow integration for broad and standardized technology integration across registries. The APIs will be integrated in the registries' workflows. In addition, working collaboratively with domain experts, the team will extend the information extraction across biomarkers and capture disease progression such as metastasis and recurrence. This pilot is engaging in several partnerships with academic and commercial entities to bring in heterogeneous data sources for more effective longitudinal trajectory modeling. Efforts to understand causal inference beyond treatment (social, economic, and environmental) impact in the real world are also part of future plans.

## Looking Ahead: Opportunities and Challenges

In addition to large-scale computing as a critical and necessary element to pursue the many opportunities for AI in cancer research, other areas must also develop to realize the tremendous potential. In this section, we list some of these opportunities.

First, HPC platforms provide high-speed interconnect between compute nodes that is integral in handling the communication for data or model parallel training. While cloud platforms have recently made significant investments in improving interconnect, this remains a challenge and would encourage projects like Pilot Three to limit distributed training to a single node. That said, on-demand nature of cloud platforms can allow for more efficient resource utilization of AI workflows, and the modern Linux environments and familiar hardware configurations available on cloud platforms offer superior support for AI workflow software which can increase productivity.

Second, the level of available data currently limits the potential for AI in cancer research. Developing data resources of sufficient size, quality, and coherence will be essential for AI to develop robust models within the domain of the available data resources.

Third, evaluation and validation of data-driven AI models, and quantifying the uncertainty in individual predictions, will continue to be an important aspect for the adoption of AI in cancer research, posing a challenge to the community to concurrently develop criteria for evaluation and validation of models while delivering the necessary data and large-scale computational resources required.

In the next two subsections, we highlight two efforts within the JDAC4C collaboration to address these challenges. The first focuses on scaling the training of the deep neural network application on HPC systems, and the second quantifies the uncertainty in the trained models to build a measure of confidence and limits on how to use them in production.

### CANDLE: Cancer Distributed Learning Environment

CANDLE (16), builds a single, scalable deep neural network application and is being used to address the challenges in each of the JDACS4C pilots.

The challenge problem for the CANDLE project is to enable the most challenging deep learning problems in cancer research to run on the most capable supercomputers in the DOE and NIH. Implementations of CANDLE have been tested on the DOE Titan, Cori, Theta and Summit systems, and using container technologies on the NIH Biowulf system (31). The CANDLE software builds on open source deep learning frameworks including Keras, TesnsorFlow and PyTorch. Through collaborations with DOE computing centers, HPC vendors and Exascale Computing Project (ECP) co-design and software technology projects, CANDLE is being prepared for the coming DOE exascale platforms.

Features currently supported in CANDLE include feature selection, hyperparameter optimization, model training, inferencing and UQ. Future release plans call for supporting experimental design, model acceleration, uncertainty guided inference, network architecture search, synthetic data generation and data modality conversion. These features have been used to evaluate over 20,000 models in a single run on a DOE HPC system.

The CANDLE project also features a set of deep learning benchmarks that are aimed at solving a problem associated with each of the pilots. These benchmarks embody different deep learning approaches to problems in cancer biology, and they are implemented in compliance with CANDLE standards making them amenable to large-scale model search and inferencing experiments.

### Uncertainty Quantification

UQ is a critical component across all three JDACS4C pilots. It is a field of analysis that estimates accuracy under multi-modal uncertainties. UQ allows detecting unreliable model predictions (32) and provides for improved design of experiments. UQ quantifies the effects of statistical fluctuations, extrapolation, overfitting, model misspecification and sampling biases, resulting in confidence measures for individual model prediction.

Historically, results from computational modeling in the biological sciences did not incorporate UQ, but measures of certainty are essential for *actionable* predictive analytics (33). The problems are exacerbated as we start addressing problems with poorly understood causal models using large—but noisy, multimodal and incomplete—data sets. Methodological advances are allowing all three pilots to use HPC technology to simultaneously estimate the uncertainty along with the results.

In addition to providing confidence intervals, the development of new UQ technology allows assessment and improvement of data quality (34); evaluation and design of models appropriate to the data quality and quantity; and prioritization of further observations or experiments that can best improve model quality. These developments are currently being tested in the JDACS4C pilots and are likely to impact the wider application of large-data-driven modeling.

## Conclusion

The JDACS4C collaboration continues to provide valuable insights into the future for AI in cancer research and the essential role that extreme-scale computing will have in shaping and informing that future. Concepts have been transformed into preliminary practice in a short period of time, as a result of multi-disciplinary teamwork and access to advanced computing resources. AI is being used to guide experimental design to make more effective use of valuable laboratory resources, to develop new capabilities for molecular simulation, and to streamline and improve efficiencies in the acquisition of clinical data.

The JDACS4C collaboration established a foundation for team science and is enabling innovation at the intersection of advanced computing technologies and cancer research. The opportunities for extreme-scale computing in AI and cancer research extend well beyond these pilots.

## Author Contributions

GZ made the paper plan, wrote the abstract, and assembled the manuscript. ES, EG, AG, TH, and CL contributed to the introduction. RS, JD, and YE made substantial contribution to the conception and design of the work in Pilot One. TBr and FX provided acquisition, analysis, and interpretation of data in Pilot One. FS and DN made substantial contributions to the conception, design, and execution of Pilot Two. GT and LP made substantial contribution in designing, developing, and writing the section about Pilot Three. RS made substantial contribution to the conception and design of the work in CANDLE. RS, TBr, TBh, and FX developed and optimized predictive models with UQ in Pilots One, Three, and CANDLE. GZ contributed to extending the application of CANDLE at NIH. ES and EG contributed to the opportunities and challenges. TBh wrote and developed the section related to UQ. EG, AG, TH, and CL contributed to the conclusion.

### Conflict of Interest

The authors declare that the research was conducted in the absence of any commercial or financial relationships that could be construed as a potential conflict of interest.
